# Real-world evidence in a large cohort comparing FAPI and FDG PET/CT for hepatopancreatobiliary cancer diagnosis

**DOI:** 10.1007/s00259-026-08112-w

**Published:** 2026-08-12

**Authors:** Suneetha Batchu, Mara M.K. Veenstra, David M. de Jong, Mohan Roop Jayanthi, Erik Vegt, Sana F. Memon, Abhishek Tyagi, Daniel Bos, Sundeep Lakhtakia, Marco J. Bruno, D. Nageshwar Reddy, Hardik Rughwani, Maarten G.J. Thomeer

**Affiliations:** 1 Department of Nuclear Medicine, Asian Institute of Gastroenterology (AIG Hospitals), Hyderabad, India; 2 Department of Radiology & Nuclear Medicine, Erasmus MC University Medical Centre Rotterdam, Rotterdam, The Netherlands; 3 Department of Gastroenterology and Hepatology, Erasmus MC University Medical Centre Rotterdam, Rotterdam, The Netherlands; 4 Department of Gastroenterology, Asian Institute of Gastroenterology (AIG Hospitals), Hyderabad, India; 5 Department of Artificial Intelligence, Asian Institute of Gastroenterology (AIG Hospitals), Hyderabad, India; 6 Department of Epidemiology, Erasmus MC University Medical Centre Rotterdam, Rotterdam, The Netherlands; 7 Rotterdam, 3015 GD The Netherlands

**Keywords:** Radionuclide Imaging, Biliary Tract Neoplasms, Liver Neoplasms, Pancreatic Neoplasms, Diagnostic Imaging, Positron Emission Tomography

## Abstract

**Purpose:**

Diagnosing hepatopancreatobiliary (HPB) malignancies remains challenging. Conventional imaging often lacks sensitivity for occult nodal and distant metastases, and FDG PET/CT has variable performance due to low uptake in several tumour subtypes and false-positive uptake in inflammation. Fibroblast activation protein inhibitor (FAPI) PET/CT has emerged as a promising alternative. This study compared the diagnostic accuracy of FDG PET/CT, FAPI PET/CT, and a dual-tracer approach in suspected HPB malignancies.

**Methods:**

This retrospective analysis included patients who underwent both [^18^F]FDG and [^68^Ga]Ga-FAPI-46 PET/CT for suspected HPB cancer, including cholangiocarcinoma, gallbladder carcinoma, pancreatic carcinoma, periampullary carcinoma, and hepatocellular carcinoma. Histopathology and/or clinical follow-up served as reference standard. FDG PET/CT classification used an SUV_max_ cut-off derived from receiver-operating characteristic analysis, while FAPI PET/CT classification followed expert clinical interpretation. Sensitivity, specificity, and accuracy metrics were compared using paired statistics.

**Results:**

176 patients were included. FAPI PET/CT demonstrated higher sensitivity than FDG PET/CT (93.0% vs. 76.5%, *p* = 0.002). Primary tumour diagnostic accuracy was 78.9% for FAPI versus 68.3% for FDG (*p* = 0.021). A dual-tracer approach did not outperform FAPI (76.7% vs. 78.9%, *p* = 0.711). For nodal disease, FAPI showed 86.8% diagnostic accuracy, compared with 71.1% for FDG (*p* = 0.004), driven by higher specificity. For distant metastases, accuracy was 83.9% for FAPI versus 80.0% for FDG (*p* = 0.625).

**Conclusion:**

FAPI PET/CT outperformed FDG PET/CT in this retrospective analysis, with dual-tracer imaging offering no additional benefits. These results show great promise for clinical application of FAPI PET/CT for the detection of HPB tumours and their metastases to be confirmed by independent prospective studies.

**Supplementary Information:**

The online version contains supplementary material available at https://doi.org/10.1007/s00259-026-08112-w.

## Background

Hepatopancreatobiliary (HPB) malignancies, including hepatocellular carcinoma (HCC), cholangiocarcinoma, gallbladder carcinoma, and pancreatic cancer, represent a heterogeneous group of aggressive malignant tumours associated with poor prognosis [1]. Accurate preoperative staging is essential to guide therapeutic strategies, particularly the decision between proceeding with a curative resection and initiating palliative treatment. While contrast-enhanced computed tomography (CT) and magnetic resonance imaging (MRI) remain the diagnostic mainstays, their sensitivity for occult nodal or distant metastases is limited [2–4].

[^18^F]fluorodeoxyglucose (FDG) positron emission tomography (PET)/CT has been adopted in several regions, particularly in Asia and the United States, to complement conventional imaging in the evaluation of HPB cancers [5, 6]. Its principal strength lies in the detection of unsuspected metastatic disease, thereby influencing management decisions and preventing non-therapeutic surgery. FDG uptake also reflects tumour glycolytic activity and may provide prognostic information. However, its diagnostic performance is heterogeneous: well-differentiated HCC, small cholangiocarcinomas, and infiltrative or mucinous subtypes often show low FDG avidity, while physiological hepatic uptake reduces lesion visibility [7–9]. Specificity is further compromised in the presence of inflammatory or infectious processes including primary sclerosing cholangitis, autoimmune cholangitis or inflammation caused by biliary stone disease of biliary stents. Consequently, FDG PET/CT is not uniformly recommended across international guidelines, and its role remains variable in routine practice [10–19].

Fibroblast activation protein inhibitor (FAPI) radiotracers have emerged as promising alternatives. FAP is overexpressed by cancer-associated fibroblasts, a hallmark of the dense desmoplastic stroma characteristic of many HPB tumours [20–22]. FAPI PET/CT has demonstrated high tumour-to-background ratios, clear lesion conspicuity in the liver, and consistently high detection rates across tumour subtypes irrespective of differentiation [23–26]. Early studies indicate that FAPI imaging may overcome several of the recognised limitations of FDG PET/CT, particularly the low sensitivity in infiltrative or small-volume disease and the confounding effect of physiological hepatic uptake [24, 27, 28]. These features position FAPI as an attractive modality for comprehensive staging of HPB cancers.

Despite these promising attributes, the evidence base for FAPI PET/CT in HPB malignancies remains limited. Most studies to date are small, heterogeneous, or lack intra-individual comparison with FDG, and many do not provide robust histopathological verification or systematic assessment for nodal and metastatic disease [29]. Consequently, the comparative diagnostic performance of FDG and FAPI PET/CT remains undetermined. To reflect routine clinical practice, where biologically distinct HPB malignancies are commonly evaluated within shared diagnostic pathways, we assessed diagnostic performance across the HPB spectrum while also performing tumour-specific subgroup analyses. We hypothesised that FAPI would provide improved lesion detection compared with FDG, particularly at the primary tumour and metastatic sites. To test this hypothesis, we performed a paired intra-individual comparison of [^18^F]FDG and [^68^Ga]Ga-FAPI-46 PET/CT imaging for suspected HPB disease prior to histopathological confirmation. In addition, by directly comparing both tracers in the same patients, this study aimed to evaluate their complementary and combined value.

## Methods

This study represents a retrospective analysis of consecutively imaged patients from an institutional clinical imaging program comparing FDG and FAPI. Although imaging was prospectively performed as part of clinical care, the present analysis was retrospective in design and was not pre-registered as a diagnostic accuracy trial. This study was performed in line with the principles of the Declaration of Helsinki. Approval was granted by the local Ethics Committee (IEC-BH&R 76), and all patients signed an informed consent form. Patients were included if they underwent both FDG and FAPI PET/CT between February 2022 and October 2024 as part of their clinical evaluation of suspected HPB malignancy, or if they underwent both scans for response-assessment in known malignancy. Additionally, patients were included if scans, which were initially performed for suspected other gastrointestinal cancer, showed findings suggestive of HPB malignancy, leading to a revised clinical suspicion of HPB disease. Malignancy types were categorised as follows: cholangiocarcinomas (subdivided into intrahepatic cholangiocarcinoma, arising from within the intrahepatic bile ducts, perihilar cholangiocarcinomas, originating in the extrahepatic bile ducts proximal to the cystic duct, and distal cholangiocarcinomas, located distal to the cystic duct in the common bile duct), gallbladder carcinomas, pancreatic carcinomas, periampullary carcinomas (tumours located around the ampulla of Vater, including the pancreatic head, distal common bile duct, and duodenum), and HCC. The inclusion criteria comprised the availability of histopathology or a minimum of six months of clinical follow-up for the reference standard. Patients with inadequate clinical records or insufficient histopathology were excluded. Clinical data potentially relevant to PET interpretation were collected, including previous oncological treatment, hepatobiliary interventions (e.g. biliary stent or drain), abdominal surgery, and underlying inflammatory or chronic hepatopancreatic conditions.

### Imaging protocol and quantitative analysis

Whole body PET images were acquired from vertex to mid-thigh using a Biograph Horizon scanner (Siemens Healthineers, Erlangen, Germany) after intravenous administration of 4–6 MBq/kg (0.12–0.15 mCi/kg) [^18^F]FDG. Data were reconstructed with contrast-enhanced CT-based attenuation correction. The next day, whole body PET/CT was performed from the vertex to the mid-thigh using 2–3 MBq/kg (0.05–0.08 mCi/kg) [^68^Ga]Ga-FAPI-46, with non-contrast-enhanced low dose CT images. To compensate for the lower administered activity of [^68^Ga]Ga-FAPI-46, PET acquisition was performed using a longer emission time per bed position (45 s/bed) than for [^18^F]FDG PET/CT (30 s/bed).

FDG and FAPI PET/CT scans were interpreted simultaneously by board-certified nuclear medicine physicians as part of routine clinical care where they had access to clinical context, including CT, MRI and endoscopy findings. PET findings were interpreted in conjunction with available anatomical imaging. As both PET/CTs were performed as part of a routine clinical workup according to local practice, pathology results were nearly always pending and unavailable for review. Both FDG and FAPI PET/CT images were evaluated side-by-side during the same reporting session to form a single integrated report. Although this reflects real-world diagnostic workflows, it introduces the possibility of interpretation bias, as knowledge of one tracer may have influenced interpretation of the other. Therefore, this study should be considered a retrospective paired cohort analysis rather than an independent blinded head-to-head diagnostic accuracy study. Images were assessed visually using a qualitative scale (non-avid, low, moderate, high uptake), and semi-quantitatively using maximum standardized uptake values (SUV_max_), consistent with clinical reporting conventions. Uptake was considered suspicious for malignancy if it exceeded background physiological activity and demonstrated a focal, non-physiological distribution with or without corresponding anatomical abnormalities on CT. Background physiological activity was measured using volumes of interest in normal liver tissue and the blood pool (thoracic aorta) to determine target-to-background ratios (TBR).

For FDG PET/CT, receiver-operating characteristic (ROC) analysis was performed to explore the diagnostic utility of SUV_max_. Because visual interpretation of FDG uptake was frequently equivocal in this cohort, particularly in case of mild to moderate uptake, which could reflect either inflammation or malignancy, an exploratory data-driven SUV_max_ threshold was required. The Youden index was therefore applied to determine the cut-off value that maximised sensitivity and specificity. Primary lesions exceeding the cut-off were considered positive; those below were classified as negative. Lymph nodes were deemed positive if they showed noticeable (mild to moderate or high) uptake above background and were suspicious for possible involvement, or, in case of equivocal findings, when histopathological correlation was recommended. Distant metastases were scored according to the reporting physician’s interpretation and also classified as positive when histopathological correlation was recommended in cases with lower diagnostic confidence. In contrast, FAPI PET/CT interpretation was generally unambiguous, as malignant uptake in primary tumour, lymph nodes, and potential distant metastases could be distinguished with high confidence by reporting physicians. Accordingly, FAPI results were classified directly according to the clinical impression documented in the original PET/CT reports, rather than based on an SUV_max_ threshold. Again, in case of diagnostic uncertainty, i.e. in lesions with low to moderate FAPI uptake that were possibly involved, reactive or inflammatory, primary lesions and possible metastases were classified as positive. FDG, FAPI, and a dual-tracer PET/CT, defined as positive for suspected malignancy when at least one individual tracer was classified as positive, were compared with histopathology or clinical follow-up as the reference standard.

### Reference standard

Histopathology or cytology was used as the primary reference standard whenever available. Pathological diagnosis was based on the guidelines of the World Health Organisation’s classification of tumours of the liver and intrahepatic bile duct, classification of tumours of the gallbladder and extrahepatic bile ducts, and classification of tumours of the pancreas [30]. If resection specimen were available following imaging, these were regarded as the primary form of histopathology reference, followed by biopsies and then cytology. In cases where tissue sampling was inconclusive, clinically suspected to be false negative, or not feasible, a composite reference standard consistent with recommendations from the European Association of Nuclear Medicine (EANM) [31] and the Society of Nuclear Medicine and Molecular Imaging (SNMMI) [32] for oncologic imaging studies was applied. This included clinical follow-up, radiologic progression, and/or biochemical markers to establish the final diagnosis. The composite reference standard was also applied to score diagnostic performance for lymph nodal and distant metastases in patients who did not have available pathology for these disease sites. A minimum follow-up of six months was required for non-biopsied lesions to be considered benign or stable. Patients with inconclusive pathology and follow-up of less than 6 months were excluded.

### Definitions of diagnostic categories

For diagnostic accuracy analysis, imaging findings were classified using a standardized 4-point framework, based on correlation with clinical, radiological, and histopathological data, as described below.

#### True positive (TP)

A scan or lesion was defined as a true positive if uptake levels were deemed suspicious for malignancy, and any of the following confirmed the malignant nature of the lesion:

- Histopathological or cytological confirmation of malignancy at the corresponding site;
- Radiologic progression of the lesion on follow-up imaging consistent with malignant growth or metastasis;

#### False positive (FP)

A scan or lesion was defined as a false positive if uptake was interpreted as suspicious, but:

- Histopathology or cytology confirmed benign pathology; or.
- Lesions demonstrated radiologic stability or resolution without oncologic treatment; or.
- No clinical or biochemical evidence of malignancy developed during follow-up.

#### True negative (TN)

A scan or lesion was defined as true negative if uptake was interpreted as non-suspicious or absent, and:

- Histopathology or cytology confirmed absence of malignancy; or.
- The lesion remained radiologically and clinically stable or regressed without oncologic intervention during follow-up.

Mild tracer uptake considered physiologic or inflammatory in nature, which was not deemed suspicious on imaging and confirmed as benign by histology or clinical course, was also classified as a true negative.

#### False negative (FN)

A scan or lesion was defined as a false negative if there was no suspicious uptake, but:

- Histopathology or cytology subsequently confirmed malignancy at that site; or.
- Follow-up demonstrated clear radiologic progression or clinical deterioration attributable to malignancy in the area of initial PET negativity.

### Statistical analysis

Demographic and clinical characteristics were summarized using numbers and percentages for categorical variables and means with standard deviations or medians with interquartile ranges for continuous variables, as appropriate. Missing data were not imputed. Cases with inconclusive PET findings due to recent interventions or metal artefacts were excluded from diagnostic accuracy calculations but were described separately as unassessable.

Comparisons between FDG and FAPI PET/CT were conducted using paired statistical tests: McNemar’s test for categorical outcomes and Wilcoxon signed-rank test for continuous variables. Sensitivity, specificity, positive predictive value (PPV), negative predictive value (NPV), and overall accuracy were calculated using 95% confidence intervals (CIs). Subgroup and sensitivity analyses were conducted using binary logistic regression models to account for potential bias related to prior histological confirmation or treatment. No correction for multiple testing was applied. Statistical significance was defined as *p* < 0.05. Analyses were performed using SPSS version 28.0.0.0 for Windows (IBM, Armonk, New York, USA).

## Results

**Fig. 1:**
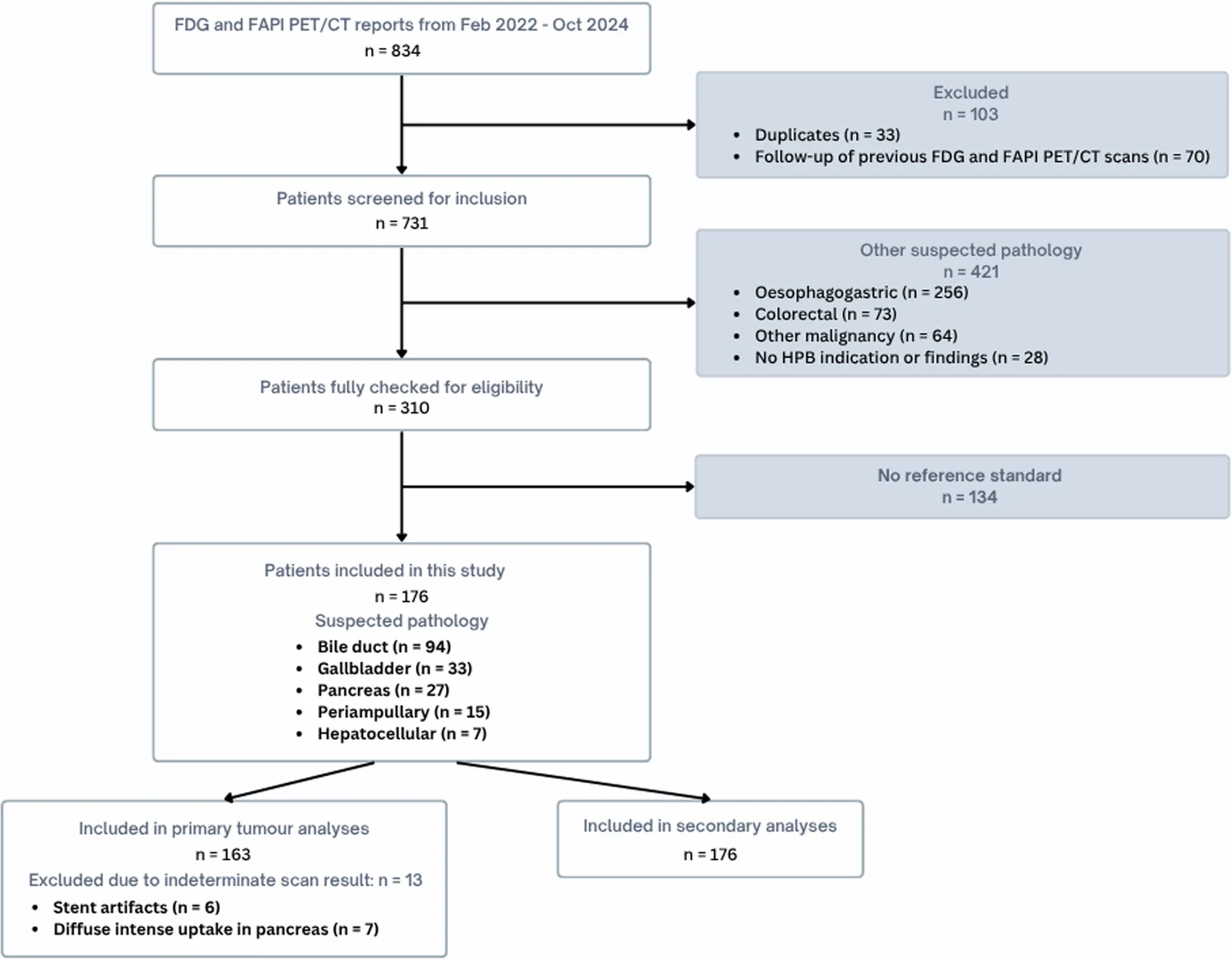
Flowchart. Shown is the patient inclusion process. The reference standard included available pathology (histology and/or cytology), or, when absent, clinical follow-up of at least three months, taking into account radiologic progression and biochemical markers to establish the final diagnosis. *HPB: hepatopancreatobiliary*

836 FDG and FAPI PET/CT were performed in 731 patients. A total of 176 patients were included in this study (Fig. 1). Most patients underwent PET/CT for the suspicion of cholangiocarcinoma (*n* = 94), followed by a suspicion of gallbladder cancer (*n* = 33), pancreatic cancer (*n* = 27), periampullary cancer (*n* = 15), and HCC (*n* = 7). Patients were mostly male (63.1%) with a median age of 56 years (interquartile range (IQR) 48–66). 126 patients (71.6%) were confirmed to have malignant disease, of whom 124 (98.4%) were proven by pathology. Baseline characteristics, including available histopathology, previous treatments and conditions potentially affecting PET interpretation, are summarised in Table 1.

**Table 1:** Baseline characteristics. Overview of patient demographics and clinical details of the study cohort. *IQR: interquartile range*

Characteristic	Total *n* = 176
**Demographics**	
Median age, years (IQR)	56 (48–66)
Sex, n (%)	
- Male	111 (63.1%)
- Female	65 (36.9%)
**Clinical context**	
Indication for PET/CT, n (%)	
- First clinical work-up of suspected disease	157 (89.2%)
- Follow-up of chronic disease	8 (4.5%)
- Response evaluation	11 (6.3%)
Prior systemic therapy, n (%)	6 (3.4%)
Prior surgical resection, n (%)	21 (11.9%)
Presence of biliary stent or drain at time of imaging, n (%)	48 (27.3%)
Known chronic liver disease or cirrhosis, n (%)	6 (3.4%)
Known chronic pancreatitis, n (%)	12 (6.8%)
**Final diagnosis**	
Malignant, n (%)	126 (71.6%)
- Cholangiocarcinoma	73 (57.9%)
- Gallbladder carcinoma	22 (17.5%)
- Pancreatic carcinoma	20 (15.9%)
- Periampullary carcinoma	8 (6.3%)
- Hepatocellular carcinoma	3 (2.4%)
Of which pathology proven, n (%)	124 (98.4%)
- Biopsy	71 (57.3%)
- Cytology	33 (26.6%)
- Resection	20 (16.1%)
Benign, n (%)	50 (28.4%)
Of which pathology proven, n (%)	48 (96.0%)
- Biopsy	24 (50.0%)
- Cytology	14 (29.2%)
- Resection	10 (20.8%)

A total of 13 patients had unassessable scan results concerning the presence of a primary tumour and were therefore excluded from primary tumour analysis. In 6 cases (all possible cholangiocarcinoma cases), the scan was considered unassessable due to the presence of a biliary stent, which obstructed the visualisation of a potential mass or introduced artifacts. In the remaining 7 cases (3 possible hilar cholangiocarcinomas and 4 possible pancreatic cancers), diffuse and intense FAPI uptake throughout the pancreas hindered the ability to assess for a possible tumour. Scoring these scans as positive would have resulted in a ‘true positive’ outcome despite indefinability for approximately half of the patients, who were found to have a malignancy. Hence, these cases were excluded from the main primary tumour analysis, but included in a supplementary primary tumour analysis (Supplementary Table 1). Unassessable primary tumour cases were included in the analysis of lymph nodes and distant metastases, where their data remain relevant.

### Tracer uptake analysis

Quantitative uptake analysis of primary tumour masses showed higher overall tracer uptake for FAPI (median SUV_max_ 15.2 [IQR 12.3–20.5]) compared with FDG (median SUV_max_ 5.5 [IQR 3.7–9.7], *p* < 0.001). Tumour uptake was higher for FAPI compared with FDG for all cancer subtypes, especially in cholangiocarcinomas (median SUV_max_ 14.6 [IQR 12.2–19.3] versus median SUV_max_ 4.7 [IQR 3.6–9.7], *p* < 0.001), reaching statistical significance for almost all tumour subtypes (Table 2). Tumour distinguishability was also better for FAPI compared with FDG, with median TBR relative to normal liver tissue being 3.3–4.5 versus 1.1–2.2 (*p* < 0.001) in primary liver tumours, and median TBR relative to the blood pool being 8.0 and 6.6 versus 2.6 and 2.1 (both statistically significant) in pancreatic carcinomas and periampullary carcinomas, respectively. FAPI showed higher uptake in confirmed pathological lymph nodes than FDG (median SUV_max_ 13.9 [IQR 9.6–20.8] versus 7.5 [4.5–11.9], *p* = 0.004), also yielding higher TBR relative to the blood pool (median TBR 5.8 [IQR 3.9–7.6] versus 3.2 [2.1-5.0], *p* = 0.004). Similar results for both tracers were found for distant metastases (Table 2).

**Table 2:** Semi-quantitative uptake analysis. Shown are the [^18^F]FDG and [^68^Ga]Ga-FAPI-46 SUV_max_ and TBR with corresponding interquartile ranges of all malignant primary and secondary tumour types. Uptake between scans was compared in all patients with proven malignancy (*n* = 126). Four patients with cholangiocarcinoma and 3 patients with pancreatic cancer were excluded from analysis of the primary tumour mass due to unassessable scan results (total *n* = 119). TBR was calculated compared with uptake in the blood pool unless the lesion was present in the liver. For liver lesions, TBR was calculated compared with uptake in normal liver tissue. * TBR relative to normal liver tissue. † Wilcoxon signed-rank test. *SUV: Standardized Uptake Value*,* TBR: Target-to-Background Ratio*,* HPB: Hepatopancreatobiliary*

		FDG SUV_max_	FAPI SUV_max_	*p*-value^†^	FDG TBR	FAPI TBR	TBR Ratio	*p*-value^†^
	**Primary tumour mass**							
	All HPB malignancies (*n* = 119)	5.5 (3.7–9.7)	15.2 (12.3–20.5)	**< 0.001**	2.2 (1.4–3.5)	6.4 (5.0-8.5)	2.9	**< 0.001**
*	Cholangiocarcinoma (*n* = 69)	4.7 (3.6–9.7)	14.6 (12.1–19.3)	**< 0.001**	1.1 (1.0-2.3)	3.3 (1.8–6.1)	3.0	**< 0.001**
*	Intrahepatic (*n* = 8)	9.9 (8.1–21.3)	17.9 (14.9–24.4)	0.161	3.5 (1.7–6.6)	5.9 (3.3–8.4)	1.7	**0.025**
*	Perihilar (*n* = 44)	4.4 (3.6–8.4)	15.4 (12.4–22.1)	**< 0.001**	1.1 (1.0-2.1)	2.8 (1.8–6.5)	2.5	**< 0.001**
	Distal (*n* = 17)	4.3 (3.4–7.9)	14.2 (8.5–16.8)	**< 0.001**	1.5 (1.3-3.0)	6.0 (3.1–8.8)	4.0	**< 0.001**
*	Gallbladder carcinoma (*n* = 22)	6.5 (3.9–9.1)	14.1 (9.8–20.3)	**< 0.001**	2.0 (1.1–2.8)	4.5 (2.8–5.9)	2.3	**< 0.001**
	Pancreatic carcinoma (*n* = 17)	5.7 (4.5–11.1)	21.4 (14.1–25.0)	**< 0.001**	2.6 (2.0-3.8)	8.0 (6.5–9.5)	3.1	**< 0.001**
	Periampullary carcinoma (*n* = 8)	6.5 (4.1–8.7)	14.5 (14.1–20.5)	**0.017**	2.1 (2.0-2.6)	6.6 (5.1–8.1)	3.1	**0.043**
*	Hepatocellular carcinoma (*n* = 3)	7.7 (5.4–10.0)	10.0 (3.0–17.0)	0.655	2.2 (2.0-2.5)	1.9 (1.0-2.8)	0.9	0.655
	**Involved lymph nodes**							
	Abdominal (*n* = 30)	6.5 (4.5–11.4)	12.7 (8.6–20.5)	**0.005**	2.5 (2.0-4.4)	5.6 (3.6–7.5)	2.2	**0.007**
	Thoracic (*n* = 3)	9.6 (8.5–16.0)	15.2 (14.1–16.1)	0.593	3.7 (3.1–7.3)	6.3 (6.0-6.7)	1.7	0.593
	Overall (*n* = 33)	7.5 (4.5–11.9)	13.9 (9.6–20.8)	**0.004**	3.2 (2.1-5.0)	5.8 (3.9–7.6)	1.8	**0.004**
	**Distant metastases**							
	Omentoperitoneal (*n* = 16)	5.4 (3.0–7.0)	10.1 (7.1–15.4)	**0.005**	2.0 (1.3–2.3)	4.0 (2.7–5.3)	2.0	**0.008**
*	Intrahepatic (*n* = 9)	10.2 (5.0-11.6)	12.2 (6.8–19.4)	0.155	2.6 (1.5–4.1)	6.6 (2.7–10.2)	2.5	**0.038**
	Overall (*n* = 25)	5.9 (3.6–10.3)	11.0 (7.0-17.4)	**0.003**	2.1 (1.6–4.2)	4.5 (2.9–6.1)	2.1	**0.005**

### Diagnostic accuracy analysis

ROC analysis for FDG SUV_max_ of the primary tumour demonstrated poor discriminatory performance, with an AUC of 0.591 (Supplementary Fig. 1). The optimal FDG SUV_max_ cut-off derived from Youden’s index was 3.65, which provided a marginal separation between malignant and non-malignant lesions. Across all HPB malignancies, FAPI demonstrated higher diagnostic accuracy compared with FDG for detection of the primary tumour mass (78.9% versus 68.3%, *p* = 0.021, Table 3). This improvement was primarily driven by a significantly higher sensitivity of FAPI (93.0% versus 76.5%, *p* = 0.002), while specificity was only moderate for both tracers where differences were not statistically significant (43.5% for FAPI versus 47.8% for FDG, *p* = 0.791). Outcomes remained similar following additional analyses including cases with unassessable scan results (Supplementary Table 1).

**Table 3:** Diagnostic accuracy of [^18^F]FDG, [^68^Ga]Ga-FAPI-46, and dual-tracer PET/CT for detecting hepatopancreatobiliary cancer. Shown are the diagnostic accuracy with their corresponding confidence intervals (CIs). The total number of patients is based on the full study cohort minus those with unassessable primary tumour findings (*n* = 176–13 = 163). Dual-tracer PET/CT was deemed positive when at least one of the scans was positive for malignancy. Green p-values represent statistically significant improvement using a dual-tracer approach compared to the single-tracer approach. Red p-values represent statistically significant decrease in performance using a dual-tracer approach compared with a single-tracer approach. P-values of tumour subgroups were non-significant due to their relatively small sample size and were omitted for clarity. *McNemar’s test. *PPV: Positive Predictive Value*,* NPV: Negative Predictive Value*,* HPB: Hepatopancreatobiliary*

	Tracer	Sensitivity (95% CI) *p*-value*	Specificity (95% CI) *p*-value*	PPV (95% CI) *p*-value*	NPV (95% CI) *p*-value*	Accuracy (95% CI) *p*-value*
Primary tumour mass						
All HPB malignancies (*n* = 163)	FDG	76.5% (68.0-83.3)	47.8% (34.1–61.9)	78.6% (70.1–85.2)	44.9% (31.9–58.7)	68.3% (60.8–75.0)
	FAPI	93.0% (86.9–96.4)	43.5% (30.2–57.8)	80.5% (72.9–86.3)	71.4% (52.9–84.8)	78.9% (71.9–84.5)
	Dual tracer strategy	99.1% (95.3–99.9)	28.9% (17.7–43.4)	78.2% (70.9–84.1)	92.9% (68.5–98.7)	79.5% (72.6–85.0)
	FDG vs. FAPI	**0.002**	0.791	**0.002**	0.791	**0.021**
	Dual vs. FDG	**< 0.001**	**0.008**	**< 0.001**	**0.008**	**0.002**
	Dual vs. FAPI	**0.008**	**0.031**	**0.008**	**0.031**	0.791
Gallbladder carcinoma (*n* = 33)	FDG	81.8% (61.5–92.7)	27.3% (9.8–56.6)	69.2% (50.0-83.5)	42.9% (15.8–75.0)	63.6% (46.6–77.8)
	FAPI	90.9% (72.2–97.5)	18.2% (5.1–47.7)	69.0% (50.8–82.7)	50.0% (15.0–85.0)	66.7% (49.6–80.3)
	Dual tracer strategy	95.5% (78.2–99.2)	0.0% (0.0-25.9)	65.6% (48.3–79.6)	0.0% (0.0-79.4)	63.6% (46.6–77.8)
Pancreatic carcinoma (*n* = 23)	FDG	86.7% (62.1–96.3)	57.1% (25.1–84.2)	81.3% (57.0-93.4)	66.7% (30.0-90.3)	77.3% (56.6–89.9)
	FAPI	100% (79.6–100)	57.1% (25.1–84.2)	83.3% (60.8–94.2)	100% (51.0-100)	86.4% (66.7–95.3)
	Dual tracer strategy	100% (79.6–100)	57.1% (25.1–84.2)	83.3% (60.8–94.2)	100% (51.0-100)	86.4% (66.7–95.3)
Periampullary carcinoma (*n* = 15)	FDG	87.5% (52.9–97.8)	42.9% (15.8–75.0)	63.6% (35.4–84.8)	75.0% (30.1–95.4)	66.7% (41.7–84.8)
	FAPI	87.5% (52.9–97.8)	57.1% (25.1–84.2)	70.0% (39.7–89.2)	80.0% (37.6–96.4)	73.3% (48.1–89.1)
	Dual tracer strategy	100% (67.6–100)	42.9% (15.8–75.0)	66.7% (39.1–86.2)	100% (43.9–100)	73.3% (48.1–89.1)
Hepatocellular carcinoma (*n* = 7)	FDG	100% (34.2–100)	50.0% (15.0–85.0)	50.0% (15.0–85.0)	100% (34.2–100)	66.7% (30.0-90.3)
	FAPI	50.0% (9.5–90.6)	100% (51.0-100)	100% (20.7–100)	80.0% (37.6–96.4)	83.3% (43.7–97.0)
	Dual tracer strategy	100% (34.2–100)	50.0% (15.0–85.0)	50.0% (15.0–85.0)	100% (34.2–100)	66.7% (30.0-90.3)

When stratified by tumour subtype, cholangiocarcinoma showed the most pronounced benefit, with FAPI achieving higher sensitivity (92.8% versus 69.6%, *p* = 0.002) and overall accuracy (81.2% versus 67.1%, *p* = 0.050) than FDG, although specificity was lower (31.3% versus 56.3%, *p* = 0.219, Table 4). Both perihilar and distal cholangiocarcinoma subgroups followed this trend, with statistically significantly higher sensitivity for FAPI (92.3% and 94.7%, respectively). FAPI’s specificity remained low to moderate (10.0% for perihilar and 60.0% for distal cholangiocarcinomas), though not reaching statistical significance when compared with FDG (*p* = 0.250 and *p* = 1.00, respectively). For intrahepatic cholangiocarcinoma, both tracers performed equally well, though sample size was limited. In other HPB tumour types, FAPI showed higher accuracy and similar or higher specificity than FDG in most subgroups, albeit without reaching statistical significance due to smaller sample sizes (Table 3). In pancreatic carcinoma, FAPI achieved a sensitivity of 100%, specificity of 57.1% and accuracy of 86.4%, compared to 86.7%, 57.1% and 77.3% for FDG. Similar trends were observed in gallbladder and periampullary carcinoma. A dual-tracer approach (combined interpretation of FAPI and FDG) significantly improved sensitivity (99.1% vs. 76.5% for FDG and 93.0% for FAPI, *p* < 0.001 and *p* = 0.008, respectively) but also significantly reduced specificity (28.9% vs. 47.8% for FDG and 43.5% for FAPI, *p* = 0.008 and *p* = 0.031) compared with FDG and FAPI alone. Overall diagnostic accuracy did not significantly improve when using a dual-tracer approach compared with FAPI alone (79.5% versus 78.9%%, *p* = 0.791). FDG showed significantly lower performance compared with both FAPI alone (*p* = 0.050) and a dual-tracer approach (*p* = 0.004).

**Table 4:** Diagnostic accuracy of [^18^F]FDG, [^68^Ga]Ga-FAPI-46, and dual-tracer PET/CT for detecting cholangiocarcinoma. Shown are the diagnostic accuracy metrics with their corresponding confidence intervals (CIs). The total number of cases is based on the number of patients with suspected biliary tract disease minus those with unassessable primary tumour findings (*n* = 94–9 = 85). Six of the excluded cases were due to the presence of a biliary stent. In the other three cases, the distal common bile duct could not be evaluated due to diffuse increased pancreatic [^68^Ga]Ga-FAPI-46 uptake. Dual-tracer PET/CT was deemed positive when at least one of the scans was positive for malignancy. Green p-values represent statistically significant improvement using a dual-tracer approach compared to the single tracer. *McNemar’s test. *PPV: Positive Predictive Value*,* NPV: Negative Predictive Value*

	Tracer	Sensitivity (95% CI) *p*-value*	Specificity (95% CI) *p*-value*	PPV (95% CI) *p*-value*	NPV (95% CI) *p*-value*	Accuracy (95% CI) *p*-value*
Primary tumour mass						
Cholangiocarcinoma (*n* = 85)	FDG	69.6% (57.9–79.2)	56.3% (33.2–76.9)	87.3% (76.0-93.7)	30.0% (16.7–47.9)	67.1% (56.5–76.1)
	FAPI	92.8% (84.1–96.9)	31.3% (14.2–55.6)	85.3% (75.6–91.6)	50.0% (23.6–76.3)	81.2% (71.6–88.1)
	Dual tracer strategy	100% (94.7–100)	25.0% (10.2–49.5)	85.2% (75.9–91.3)	100% (51.0-100)	85.9% (76.9–91.7)
	FDG vs. FAPI	**0.002**	0.219	**0.002**	0.219	**0.050**
	Dual vs. FDG	-	0.063	**< 0.001**	0.063	**0.002**
	Dual vs. FAPI	-	1.000	0.063	1.000	0.219
- Perihilar (*n* = 53)	FDG	69.2% (53.6–81.4)	40.0% (16.8–68.7)	81.8% (65.6–91.4)	25.0% (10.2–49.5)	63.3% (49.3–75.3)
	FAPI	92.3% (79.7–97.4)	10.0% (1.8–40.4)	80.0% (66.2–89.1)	25.0% (4.6–69.9)	75.5% (61.9–85.4)
	Dual tracer strategy	100% (91.0-100)	9.3% (1.62–37.7)	85.2% (66.4–88.5)	100% (20.7–100)	80.3% (67.0-88.8)
	FDG vs. FAPI	**0.035**	0.250	**0.035**	0.250	0.238
	Dual vs. FDG	**-**	0.250	**0.001**	0.250	**0.035**
	Dual vs. FAPI	-	1.000	0.250	1.000	0.250
- Distal (*n* = 24)	FDG	57.9% (36.3–76.9)	80.0% (37.6–96.4)	91.7% (64.6–98.5)	33.3% (13.8–60.9)	62.5% (42.7–78.8)
	FAPI	94.7% (75.4–99.1)	60.0% (23.7–88.2)	90.0% (69.9–97.2)	75.0% (30.1–95.4)	87.5% (69.0-95.7)
	Dual tracer strategy	100% (83.2–100)	40.0% (11.8–76.9)	86.4% (66.7–95.3)	100% (34.2–100)	87.5% (69.0-95.7)
	FDG vs. FAPI	**0.039**	1.000	**0.039**	1.000	0.146
	Dual vs. FDG	**-**	0.500	**0.008**	0.500	0.109
	Dual vs. FAPI	-	1.000	1.000	1.000	1.000
- Intrahepatic (*n* = 8)	FDG	100% (67.6–100)	-	100% (67.6–100)	-	100% (67.6–100)
	FAPI	100% (67.6–100)	-	100% (67.6–100)	-	100% (67.6–100)
	Dual tracer strategy	100% (67.6–100)	-	100% (67.6–100)	-	100% (67.6–100)

Full contingency tables are shown in Supplementary Tables 2–3. Overall, FDG PET/CT yielded 24 false-positive primary tumours compared with 26 for FAPI PET/CT. For both tracers, false-positive findings were most frequently associated with inflammatory conditions, including non-specific inflammation (FDG: *n* = 10; FAPI: *n* = 8), possible inflammatory bile duct strictures (*n* = 4 vs. *n* = 5), pancreatitis (*n* = 4 vs. *n* = 5), and xanthogranulomatous cholecystitis (*n* = 3 vs. *n* = 4). Less common causes included Mirizzi syndrome (*n* = 1 each), IgG4-related disease (*n* = 1 each), and ampullary adenoma (*n* = 1 each). One additional false-positive case on FAPI PET/CT occurred in a patient with recurrent pyogenic cholecystitis.

**Fig. 2:**
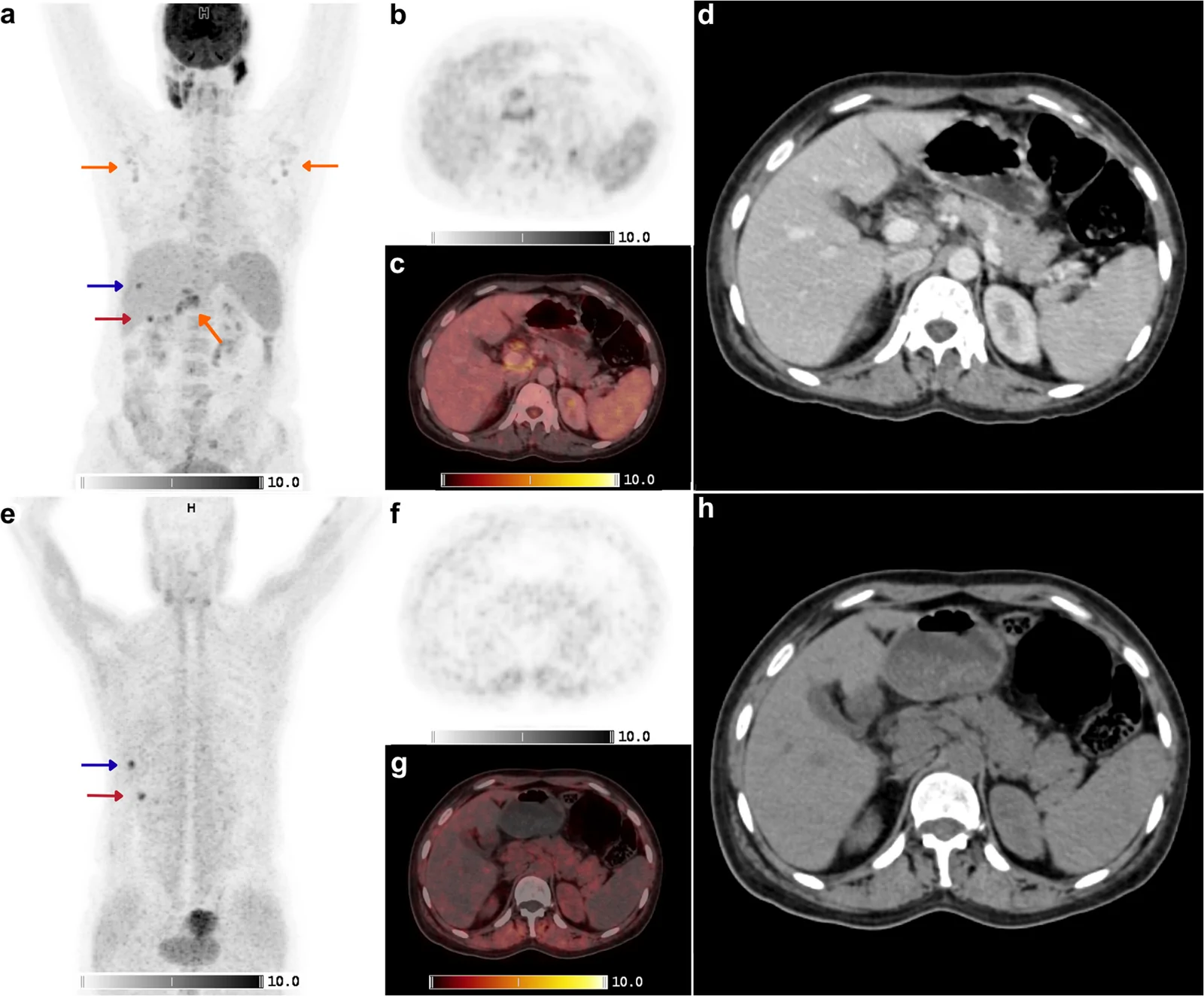
A case of reactive lymph nodes. Shown is a 38-year-old woman with histologically proven gall bladder cancer (red arrow) with a single intrahepatic metastasis (blue arrow) that are visible on both scans. FDG PET/CT showed multiple FDG avid perihilar and axillary lymph nodes (orange arrows) without corresponding FAPI avidity. Subsequent biopsy of the perihilar lymph node revealed inflammatory lymph nodes. A luteal ovarian cyst with FAPI avidity was also noted. **a**-**d**: [^18^F]FDG PET/CT, **e**-**h**: [^68^Ga]Ga-FAPI-46 PET/CT. **a**, **b**, **e**, **f**: Maximum intensity projections, **c**, **g**: axial fusion images, **d**: contrast-enhanced computed tomography, **h**: non-contrast enhanced computed tomography

Data on nodal and distant metastatic disease were available for 76 and 30 patients, respectively, with histopathology present in 59 (77.6%, of which 28 patients with biopsy, 22 resection and 9 cytology) and 12 (40.0%, 8 patients with biopsy and 4 cytology) cases. The composite reference standard was used in 17 (22.4%) and 18 (60.0%) cases. FAPI demonstrated higher overall accuracy than FDG in these secondary tumour localisations (per patient analysis, Table 5). FAPI outperformed FDG for lymph node assessment (accuracy 86.8% versus 71.1%, *p* = 0.004), mainly due to markedly higher specificity (97.2% versus 69.4%, *p* = 0.002) (Fig. 2). For distant metastases, FAPI achieved a higher detection rate (92.3% versus 80.8%), though not reaching statistical significance due to a small sample size. For lymph node staging, FDG PET/CT yielded 11 false-positive cases, all attributable to reactive lymph nodes, whereas FAPI PET/CT showed only one false-positive lymph node in a patient with chronic calcific pancreatitis. For peritoneal metastases, FAPI PET/CT was false-positive in two patients: one with perihepatic uptake following recent completion cholecystectomy and one with xanthogranulomatous cholecystitis mimicking peritoneal tumour deposits. FDG PET/CT demonstrated one false-positive peritoneal finding, also in the patient who had recently undergone completion cholecystectomy. FDG PET/CT missed distant metastases in five patients, whereas FAPI PET/CT was false-negative in two patients, both of whom were also missed by FDG PET/CT. When limiting the analysis to pathology-proven cases, FAPI continued to statistically significantly outperform FDG for lymph nodal metastasis detection. For distant metastases, however, the very limited number of histologically verified cases produced unstable accuracy estimates. Detailed results are provided in Supplementary Table 4.

**Table 5:** Diagnostic accuracy of [^18^F]FDG, [^68^Ga]Ga-FAPI-46, and dual-tracer PET/CT for detecting lymph nodal and distant metastases. Shown are the diagnostic accuracy metrics with their corresponding confidence intervals (CIs). Dual-tracer PET/CT was deemed positive when at least one of the scans was positive for malignancy. Red p-values represent a statistically significant decrease in diagnostic performance when using a dual-tracer approach compared to a single-tracer approach. Per patient analysis. *McNemar’s test. *PPV: Positive Predictive Value*,* NPV: Negative Predictive Value*

	Tracer	Sensitivity (95% CI) *p*-value*	Specificity (95% CI) *p*-value*	PPV (95% CI) *p*-value*	NPV (95% CI) *p*-value*	Accuracy (95% CI) *p*-value*
**Involved lymph nodes (** ***n*** ** = 76)**	FDG	72.5% (57.2–83.9)	69.4% (53.1–82.0)	72.5% (57.2–83.9)	69.4% (53.1–82.0)	71.1% (60.0–80.0)
	FAPI	77.5% (62.5–87.7)	97.2% (85.8–99.5)	96.9% (84.3–99.4)	79.6% (65.5–88.8)	86.8% (77.4–92.7)
	Dual tracer strategy	82.5% (68.0-91.3)	69.4% (53.1–82.0)	75.0% (60.6–85.4)	78.1% (61.2–89.0)	76.3% (65.6–84.5)
	FDG vs. FAPI	0.687	**0.002**	0.687	**0.002**	**0.004**
	Dual vs. FDG	0.125	1.000	0.125	1.000	0.125
	Dual vs. FAPI	0.500	**0.002**	0.500	**0.002**	**0.039**
**Distant metastases** (*n* = 30)	FDG	80.8% (62.1–91.5)	75.0% (30.1–95.4)	95.5% (78.2–99.2)	37.5% (13.7–69.4)	80.0% (62.7–90.5)
	FAPI	92.3% (75.9–97.9)	40.0% (11.8–76.9)	88.9% (71.9–96.1)	50.0% (15.0–85.0)	83.9% (67.4–92.9)
	Dual tracer strategy	92.3% (75.9–97.9)	50.0% (15.0–85.0)	92.3% (75.9–97.9)	50.0% (15.0–85.0)	86.7% (70.3–94.7)
	FDG vs. FAPI	0.250	1.000	0.250	1.000	0.625
	Dual vs. FDG	0.250	1.000	0.250	1.000	0.625
	Dual vs. FAPI	1.000	1.000	1.000	1.000	1.000

### Concordance and discordance

**Fig. 3:**
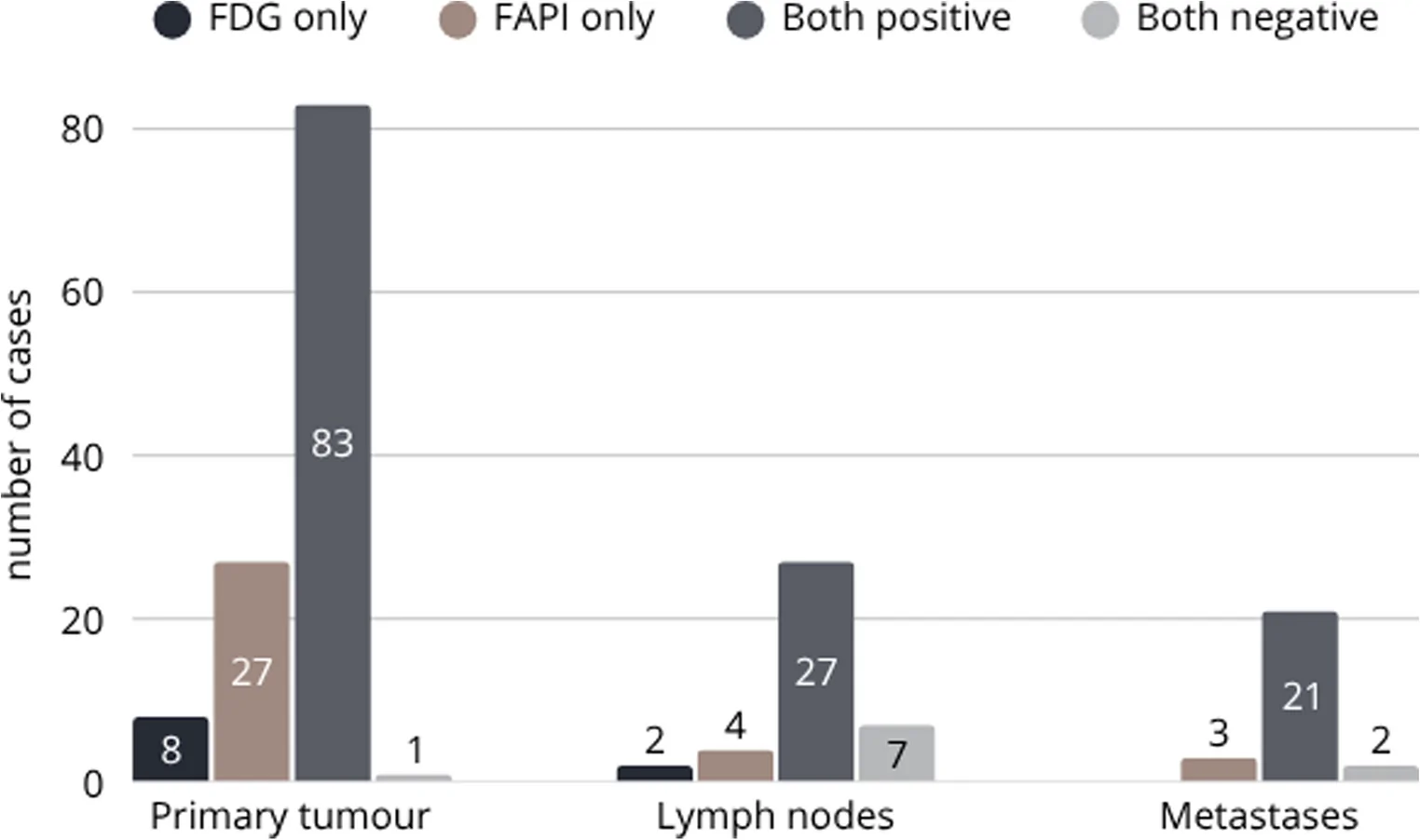
Concordance and discordance between [^18^F]FDG and [^68^Ga]Ga-FAPI-46 PET/CT. Shown are the number of concordant and discordant cases between [^18^F]FDG and [^68^Ga]Ga-FAPI-46 PET/CT in patients with confirmed malignancy. Most primary tumour masses, involved lymph nodes, and distant metastases were visualised on both scans. [^68^Ga]Ga-FAPI-46 PET/CT identified a greater number of malignant lesions that were missed by [^18^F]FDG PET/CT than vice versa and more frequently resulted in correct upstaging of disease extent. One gallbladder carcinoma was negative on both scans. Involved lymph nodes were found in the resection specimen of seven patients who had negative PET/CT findings. In two cases, small peritoneal metastases were missed on both scans but found during subsequent surgery

**Fig. 4:**
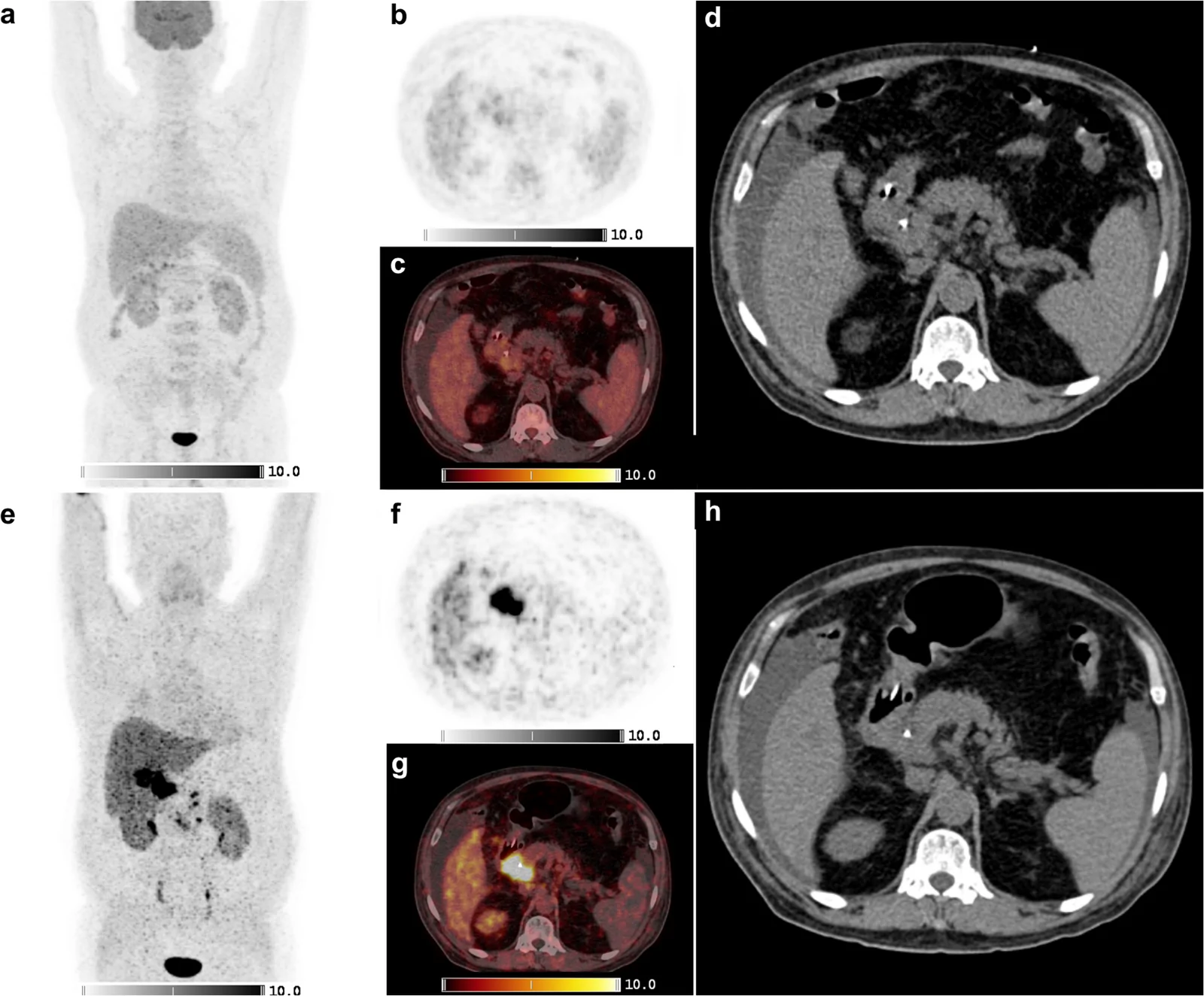
Patient with cholangiocarcinoma. Shown is a 69-year-old man with a cholangiocarcinoma in the distal common bile duct. [^18^F]FDG PET/CT was negative where [^68^Ga]Ga-FAPI-46 PET/CT showed high focal FAPI uptake in the liver hilum and surrounding lymph nodes. **a**-**d**: [^18^F]FDG PET/CT, **e**-**h**: [^68^Ga]Ga-FAPI-46 PET/CT. **a**, **b**, **e**, **f**: Maximum intensity projections, **c**, **g**: axial fusion images, **d**, **h**: non-contrast enhanced computed tomography

**Fig. 5:**
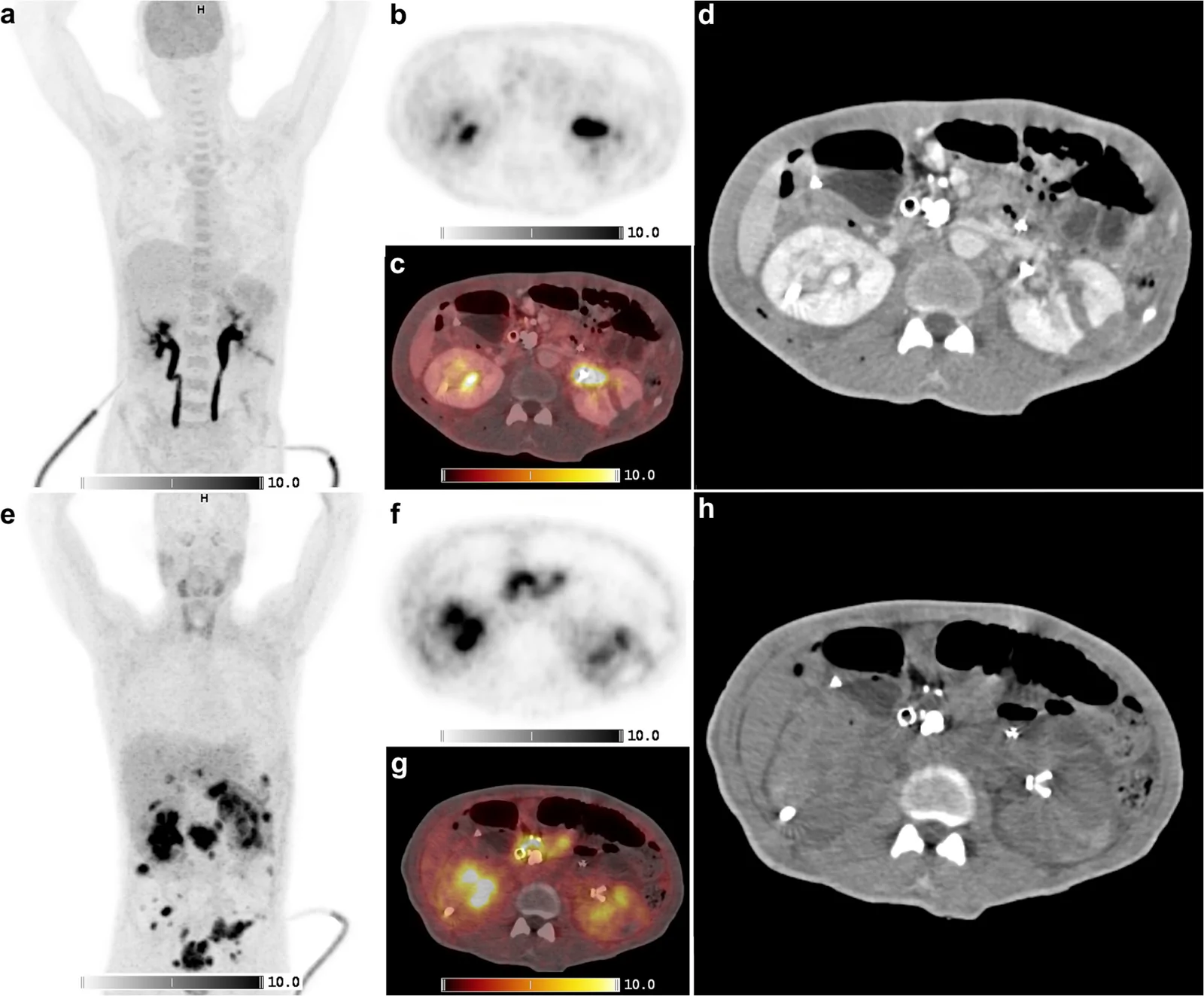
Pancreatic adenocarcinoma with peritoneal metastases. Shown is a 48-year-old man with pathologically proven metastasised pancreatic cancer. [^18^F]FDG PET/CT was negative, whilst [^68^Ga]Ga-FAPI-46 PET/CT showed high FAPI uptake in both primary and metastatic tumour locations. **a**-**d**: [^18^F]FDG PET/CT, **e**-**h**: [^68^Ga]Ga-FAPI-46 PET/CT. **a**, **b**, **e**, **f**: Maximum intensity projections, **c**, **g**: axial fusion images, **d**: contrast-enhanced computed tomography, **h**: non-contrast enhanced computed tomography

The total number of concordant and discordant FDG and FAPI PET/CT are shown in Fig. 3. Both PET/CT scans were positive in the majority of cancer patients with a primary tumour. FAPI identified 27 patients in whom FDG was negative (Fig. 4). Conversely, FDG was positive in 8 cases where FAPI showed no uptake. FAPI therefore detected 16% more primary tumours than FDG. One gallbladder carcinoma was missed on both scans. FAPI PET/CT upstaged both nodal (N-stage) and distant (M-stage) metastasis more frequently, identifying additional cases that were missed by FDG (Fig. 5).

### Subgroup analysis

Logistic regression analysis did not identify statistically significant predictors of a positive FDG PET/CT scan, although previous surgical resection showed a borderline non-significant association with reduced FDG positivity (OR 0.35, 95% CI 0.12–1.01, *p* = 0.052) (Supplementary Table 5). For FAPI, no patient or disease characteristic showed a significant association with a positive scan, except for chronic underlying liver disease, which was associated with a substantially lower likelihood of FAPI positivity (OR 0.06, 95% CI 0.06–0.54, *p* = 0.013).

False-positive analyses showed that biliary drainage was associated with increased false-positive rates for both FDG and FAPI (OR 2.99 [*p* = 0.012] and OR 2.60 [*p* = 0.025], respectively), and previous surgical resection was associated with higher false-positives for FAPI (OR 4.00, *p* = 0.013) (Supplementary Table 6). Other factors, including chronic liver disease, chronic pancreatitis, and prior chemotherapy, were not significantly associated with false-positive results. False-negative rates were generally low and not clearly associated with any patient characteristic (Supplementary Table 7). Overall, while some clinical factors were linked to modest changes in false-positive or positive scan likelihood, no single factor fully explained the occurrence of false-positive or false-negative findings.

## Discussion

This large, single-centre retrospective comparative study suggests that FAPI PET/CT achieves significantly higher diagnostic accuracy compared with FDG PET/CT for the detection and staging of hepatopancreatobiliary (HPB) malignancies. FAPI showed higher tumour-to-background ratios, clearer lesion delineation, and improved sensitivity across all major HPB tumour subtypes, particularly cholangiocarcinoma. These findings support growing evidence that FAPI can overcome limitations of FDG, which include low uptake in well-differentiated or mucinous tumours and reduced detectability of lesions adjacent to high physiological background activity such as the liver [7–9]. By identifying 16% more primary cancers than FDG and outperforming FDG for the detection of lymph nodal and distant metastases, FAPI PET/CT could potentially improve non-invasive cancer staging, and, ultimately, clinical decision making.

### Comparison with prior studies

Although several studies have demonstrated favourable FAPI uptake characteristics, most have been small and heterogeneous, with limited pathological confirmation or assessment of metastatic disease [29]. Henrar et al. reported high FAPI uptake in a comprehensive systematic review and meta-analysis of numerous smaller studies of various hepatopancreatobiliary malignancies, with SUV_max_ values typically exceeding those of FDG and producing clear lesion-to-liver contrast [29]. Our results align with these findings. As in earlier studies [33–38], FAPI also often revealed lesions with minimal or absent FDG uptake. The diagnostic accuracy observed in this study compares favourably with published performance metrics for conventional imaging modalities. Contrast-enhanced CT and MRI remain the standard for anatomical assessment but have reported sensitivities for lymph node and distant metastasis detection in the range of 30–70% [2, 39–41]. In contrast, FAPI PET/CT achieved accuracies exceeding 80% across nodal and distant metastatic sites, suggesting potential as a comprehensive staging tool.

### Interplay between FAPI and FDG in clinical interpretation

FDG and FAPI PET/CT scans were interpreted simultaneously. This approach reflects clinical reality but could bias results towards the more visually convincing tracer. Lymph nodes with diffuse or low-grade FDG uptake were frequently interpreted using FAPI results. In many cases, the absence of FAPI uptake supported a benign interpretation (e.g. reactive or inflammatory lymph nodes), while concordant FAPI uptake raised suspicion for metastasis. ROC analysis confirmed poor FDG discrimination between benign and malignant primary tumours (AUC close to 0.50), consistent with its known limitations in HPB tumours, where background uptake in inflammatory or cholestatic tissue often obscures tumour-specific signal [7–9]. While the cut-off value of 3.65 provided a formal statistical reference, its clinical relevance is questionable and may have introduced optimism bias. This underscores FAPI’s diagnostic clarity and the reduced ambiguity it offers compared with FDG.

### Limitations in inflammatory conditions

While FAPI PET/CT offered clear diagnostic advantages, there are limitations in certain cases. A total of 13 patients were excluded from the primary tumour analysis due to unassessable scan results caused by the presence of a biliary stent or diffuse FAPI uptake in cases of pancreatitis. These are clinically relevant, as it highlights a key limitation in routine practice. Many patients require prompt biliary stenting to relieve symptoms and often experiencing iatrogenic or obstructive inflammation, during which diagnostic imaging is frequently acquired. In addition, the specificity of FAPI for primary tumours was relatively low in the current study, with false positive findings most commonly associated with inflammatory changes. This may reflect increased uptake in cases of chronic inflammation or following procedures such as stent placement or surgery, but could also reflect a learning curve associated with the clinical introduction of FAPI PET/CT. In several false-positive cases, low-grade or equivocal FAPI uptake prompted recommendations for histopathological confirmation out of diagnostic caution, even when the suspicion of malignancy was modest. With increasing reader experience and growing familiarity with physiological and inflammatory uptake patterns, interpretive accuracy is expected to improve, reducing the rate of unnecessary follow-up procedures and enhancing overall specificity. Nonetheless, there is a need for further refinement of FAPI imaging protocols, especially in the pancreas, where inflammatory changes such as active pancreatitis can mimic malignancy and complicate or disable interpretation altogether.

### Dual-tracer strategy: Not superior to FAPI alone

The latest SNMMI/EANM procedure standards for oncologic PET imaging reaffirm FDG’s central role in oncologic imaging but acknowledge its limitation in low-glycolytic tumours [42]. Given the complementary nature of FDG and FAPI PET/CT, a dual-tracer strategy was considered as a potential method to improve diagnostic accuracy. This approach has shown promise for HCC using different radiotracers, but there is a significant gap in research regarding its use in cholangiocarcinomas and pancreatic cancers [43, 44]. Our study results indicated that a dual-tracer approach, although reaching significantly higher sensitivity, likely does not provide clinically meaningful value compared to FAPI alone. The significant decrease in specificity, combined with additional patient burden, cost and required infrastructure, could make a dual-tracer strategy unappealing for routine clinical implementation. While the combination of tracers may offer additional biological insights, particularly in complex cases, FAPI alone demonstrated sufficient sensitivity and tumour conspicuity to guide clinical decision-making. This suggests that, at least in this cohort, FAPI PET/CT alone is a highly effective imaging tool for HPB malignancies, without the need for FDG as an adjunct. This will likely also improve cost-effectiveness.

### Clinical implications and future directions

Integrating FAPI PET/CT into the diagnostic work-up of HPB cancers may improve preoperative staging and treatment planning. Preoperative endoscopic ultrasound (EUS) is shown to be a valuable tool for lymph node staging of cholangiocarcinomas [45]. Lymph nodal FAPI uptake could help guide targeted EUS with tissue acquisition, reducing the need for comprehensive individual lymph node assessments. The ability to accurately identify both primary and metastatic disease could also reduce the rate of futile surgery, optimise patient selection for resection or systemic therapy, and may refine radiotherapy planning by better delineating tumour margins. Dual-tracer imaging may still hold value in specific contexts, such as evaluating tumour heterogeneity or treatment response, but FAPI alone already provides substantial clinical utility. Future prospective studies should investigate tumour-specific imaging characteristics, including associations between tracer uptake, histopathological features, and their impact on staging, treatment selection, and patient management. Additionally, dynamic PET acquisition may be able to help distinguish between benign and malignant disease [46]. This technique warrants further investigation in this patient population, especially since uptake in inflammatory tissues seems to be a common limitation of most radiopharmaceuticals.

### Strengths and limitations

This study represents the largest single-centre cohort comparing FAPI and FDG PET/CT in patients with suspected or confirmed HPB malignancies, providing substantial statistical power and clinical relevance. By evaluating both tracers within the same patient cohort and imaging timeframe, direct intra-individual comparison was possible, minimising interpatient variability and increasing the reliability of the results. The inclusion of primary tumours, lymph node involvement, and distant metastases offers a comprehensive assessment of diagnostic performance across primary tumours, lymph node and distant metastases.

However, this study also has several limitations. First, although FDG and FAPI PET/CT were performed in the same patients, image interpretation was conducted in a side-by-side manner as part of routine clinical reporting. This approach reflects real-world diagnostic workflows but introduces a risk of incorporation and interpretation bias, as knowledge of one tracer may have influenced the assessment of the other. Therefore, the present study should be regarded as a retrospective paired cohort analysis rather than an independent blinded head-to-head comparison. Second, no formal interobserver agreement analysis could be performed, as all PET/CT scans were interpreted during routine clinical care by various experienced nuclear medicine physicians. While this may have introduced variability in visual scoring, it reflects real-world clinical practice and could strengthen the external validity of the results. Third, the FDG SUV_max_-based classification was derived from an exploratory ROC analysis. This threshold was not externally validated and may be subject to optimism bias, limiting its generalisability. In contrast, FAPI interpretation was based on qualitative clinical assessment, reflecting routine reporting practice but limiting strict quantitative comparability between tracers. Fourth, potential reader confirmation bias cannot be ruled out, as the interpreting nuclear medicine physicians may have been aware of prior clinical or histological findings when evaluating scans. These chances are very small, however, as new patients at our institution undergo a full diagnostic work-up within a few days, and biopsy results generally take 7–10 days to come back. Fifth, not all patients had conclusive histopathology or resection specimens available. While a substantial proportion of cases had pathological verification, particularly for primary tumours, lymph node involvement and distant metastases were partly assessed using a composite reference standard including imaging follow-up and clinical course. This may have introduced misclassification bias. However, sensitivity analyses restricted to pathology-proven cases yielded consistent results. Sixth, FAPI PET/CT showed false-positive uptake in inflammatory and post-interventional conditions, particularly in patients with biliary stents, pancreatitis and post-surgical changes. In addition, 13 cases were considered unassessable due to diffuse pancreatic uptake or artefacts, highlighting important limitations of FAPI imaging in hepatobiliary disease. Lastly, the study cohort was clinically heterogeneous, including different HPB tumour entities as well as patients with prior treatments. Although subgroup analyses were performed, some comparisons were limited by small sample sizes. Despite these limitations, the paired design and relatively large cohort provide clinically relevant comparative data, which should be validated in prospective, blinded multicentre studies.

## Conclusion

In this retrospective paired cohort analysis, FAPI PET/CT demonstrated higher sensitivity and diagnostic accuracy than FDG PET/CT across hepatopancreatobiliary malignancies. These results indicate FAPI PET/CT could potentially complement conventional imaging for comprehensive staging of HPB cancers. However, interpretation bias due to non-blinded side-by-side reading and the retrospective design limit definitive conclusions. Prospective blinded multicentre trials are warranted to confirm these findings.

## Supplementary Information

Below is the link to the electronic supplementary material.

Supplementary Material 1

## Data Availability

The datasets generated during and/or analysed during the current study are available from the corresponding author on reasonable request.
