# Supplementary material for: Real-world evidence in a large cohort comparing FAPI and FDG PET/CT for hepatopancreatobiliary cancer diagnosis
Source: Eur J Nucl Med Mol Imaging. 2026 Aug 12;53(13):8246–61. doi: 10.1007/s00259-026-08112-w (PMC13727803; doi:10.1007/s00259-026-08112-w)
Supplement: Supplementary file 1 [file 259_2026_8112_MOESM1_ESM.docx]

**Supplementary Data File**

European Journal of Nuclear Medicine and Molecular Imaging

**Real-World Evidence in a Large Cohort Supporting FAPI PET/CT as a Preferred Modality over FDG PET/CT for Hepatopancreatobiliary Cancer Diagnosis**

Suneetha Batchu^1^ and Mara M.K. Veenstra^2^, David M. de Jong^3^, Mohan Roop Jayanthi^1^, Erik Vegt^2^, Sana F. Memon^4^, Abhishek Tyagi^5^, Daniel Bos^2,6^, Sundeep Lakhtakia^4^, Marco J. Bruno^3^, D. Nageshwar Reddy^4^, Hardik Rughwani^4^ and Maarten G.J. Thomeer^2^

1. Department of Nuclear Medicine, Asian Institute of Gastroenterology (AIG Hospitals), Hyderabad, India
2. Department of Radiology & Nuclear Medicine, Erasmus MC University Medical Centre Rotterdam, Rotterdam, The Netherlands
3. Department of Gastroenterology and Hepatology, Erasmus MC University Medical Centre Rotterdam, Rotterdam, The Netherlands
4. Department of Gastroenterology, Asian Institute of Gastroenterology (AIG Hospitals), Hyderabad, India
5. Department of Artificial Intelligence, Asian Institute of Gastroenterology (AIG Hospitals), Hyderabad, India
6. Department of Epidemiology, Erasmus MC University Medical Centre Rotterdam, Rotterdam, The Netherlands

Corresponding author: Mara M.K. Veenstra, e-mail: [m.m.k.veenstra@erasmusmc.nl](mailto:m.m.k.veenstra@erasmusmc.nl)

|  | **Tracer** | **Sensitivity (95% CI)**  **p-value*** | **Specificity (95% CI)**  **p-value*** | **PPV (95% CI)**  **p-value*** | **NPV (95% CI)**  **p-value*** | **Accuracy (95% CI)**  **p-value*** |
| --- | --- | --- | --- | --- | --- | --- |
| **Primary tumour mass** |  |  |  |  |  |  |
| All HPB malignancies (n=176) | FDG | 77.1% (68.8-83.6) | 44.2% (31.6-57.7) | 76.4% (68.2-83.1) | 45.1% (32.3-58.6) | 67.2% (60.0-73.8) |
|  | FAPI | 92.6% (86.6-96.1) | 38.5% (26.5-52.0) | 77.9% (70.5-83.9) | 69.0% (50.8-82.7) | 76.4% (69.6-82.1) |
|  | Dual tracer strategy | 99.2% (95.5-99.9) | 25.5% (15.6-38.9) | 76.3% (69.1-82.2) | 92.9% (68.5-98.7) | 77.6% (70.8-83.2) |
|  | FDG vs FAPI | **0.003** | 0.607 | **0.003** | 1.000 | **0.036** |
|  | Dual vs FDG | **<0.001** | **0.004** | **<0.001** | **0.004** | **0.003** |
|  | Dual vs FAPI | **0.004** | **0.031** | **0.004** | **0.031** | 0.607 |
| Cholangiocarcinoma (n=94) | FDG | 69.9% (58.6-79.2) | 42.9% (24.5-63.5) | 81.0% (69.6-88.8) | 29.0% (16.1-46.6) | 63.8% (53.8-72.8) |
|  | FAPI | 91.8% (83.2-96.2) | 23.8% (10.6-45.1) | 80.7% (71.0-87.8) | 45.5% (21.3-72.0) | 76.6% (67.1-84.0) |
|  | Dual tracer strategy | 100% (95.0-100) | 19.0% (7.7-40.0) | 81.1% (71.8-87.9) | 100% (51.0-100) | 81.9% (72.9-88.4) |
|  | FDG vs FAPI | **0.004** | 0.219 | **0.004** | 0.219 | 0.058 |
|  | Dual vs FDG | **-** | 0.063 | **<0.001** | 0.063 | **0.002** |
|  | Dual vs FAPI | - | 1.000 | **0.031** | 1.000 | 0.125 |
| Pancreatic carcinoma (n=27) | FDG | 88.9% (67.2-96.9) | 62.5% (30.6-86.3) | 84.2% (62.4-94.5) | 71.4% (35.9-91.8) | 80.8% (62.1-91.5) |
|  | FAPI | 100% (82.4-100) | 50.0% (21.5-78.5) | 81.8% (61.5-92.7) | 100% (51.0-100) | 84.6% (66.5-93.9) |
|  | Dual tracer strategy | 100% (82.4-100) | 50.0% (21.5-78.5) | 81.8% (61.5-92.7) | 100% (51.0-100) | 84.6% (66.5-93.9) |

**Supplementary Table 1: Diagnostic accuracy calculations with the inclusion of indeterminate cases.** Shown are the diagnostic accuracy with their corresponding confidence intervals (CIs). The total number of patients is based on the full study cohort (n = 176), including those with indeterminate scan results. Indeterminate scan results were classified as positive in FAPI cases with high pancreatic uptake due to active pancreatitis. Indeterminate scan results due to the presence of a stent were classified as positive for both radiotracers, as increased uptake along the stent was observed in all cases. Dual-tracer PET/CT was deemed positive when at least one of the scans was positive for malignancy. Green p-values represent statistically significant improvement using a dual-tracer approach compared to the single-tracer approach. Red p-values represent a statistically significant decrease in performance using a dual-tracer approach compared with a single-tracer approach. P-values of the pancreatic carcinoma subgroup were non-significant due to their relatively small sample size and were omitted for clarity. *McNemar’s test. *PPV: Positive Predictive Value, NPV: Negative Predictive Value, HPB: Hepatopancreatobiliary.*

| **Tumour Location** | **Radiotracer** | | | | | |
| --- | --- | --- | --- | --- | --- | --- |
|  | **FDG** | | **FAPI** | | **Dual tracer** | |
| **All HPB Tumours** (n=163) | **Cancer** | **Benign** | **Cancer** | **Benign** | **Cancer** | **Benign** |
| **Positive PET/CT** | TP n=88 (54.0%) | FP n=24 (14.7%) | TP n=107 (65.6%) | FP n=26 (16.0%) | TP n=115 (70.6%) | FP n=32 (19.6%) |
| **Negative PET/CT** | FN n=28 (17.2%) | TN n=21 (12.9%) | FN n=9 (5.5%) | TN n=19 (11.7%) | FN n=1 (0.6%) | TN n=13 (8.0%) |
|  | **FDG** | | **FAPI** | | **Dual tracer** | |
| **Cholangiocarcinoma** (n=85) | **Cancer** | **Benign** | **Cancer** | **Benign** | **Cancer** | **Benign** |
| **Positive PET/CT** | TP n=48 (56.5%) | FP n=7 (8.2%) | TP n=64 (75.3%) | FP n=11 (12.9%) | TP n=69 (81.2%) | FP n=12 (14.1%) |
| **Negative PET/CT** | FN n=21 (24.7%) | TN n=9 (10.6%) | FN n=5 (5.9%) | TN n=5 (5.9%) | FN n=0 (0.0%) | TN n=4 (4.7%) |
|  | **FDG** | | **FAPI** | | **Dual tracer** | |
| **Gallbladder carcinoma** (n=33) | **Cancer** | **Benign** | **Cancer** | **Benign** | **Cancer** | **Benign** |
| **Positive PET/CT** | TP n=18 (54.5%) | FP n=8 (24.2%) | TP n=20 (60.6%) | FP n=9 (27.3%) | TP n=21 (63.6%) | FP n=11 (33.3%) |
| **Negative PET/CT** | FN n=4 (12.1%) | TN n=3 (9.1%) | FN n=2 (6.1%) | TN n=2 (6.1%) | FN n=1 (3.0%) | TN n=0 (0.0%) |
|  | **FDG** | | **FAPI** | | **Dual tracer** | |
| **Pancreatic carcinoma** (n=23) | **Cancer** | **Benign** | **Cancer** | **Benign** | **Cancer** | **Benign** |
| **Positive PET/CT** | TP n=13 (56.5%) | FP n=3 (13.0%) | TP n=15 (65.2%) | FP n=3 (13.0%) | TP n=15 (65.2%) | FP n=3 (13.0%) |
| **Negative PET/CT** | FN n=2 (8.7%) | TN n=4 (17.4%) | FN n=0 (0.0%) | TN n=4 (17.4%) | FN n=0 (0.0%) | TN n=4 (17.4%) |
|  | **FDG** | | **FAPI** | | **Dual tracer** | |
| **Periampullary carcinoma** (n=15) | **Cancer** | **Benign** | **Cancer** | **Benign** | **Cancer** | **Benign** |
| **Positive PET/CT** | TP n=7 (46.7%) | FP n=4 (26.7%) | TP n=7 (46.7%) | FP n=3 (20.0%) | TP n=8 (53.3%) | FP n=4 (26.7%) |
| **Negative PET/CT** | FN n=1 (6.7%) | TN n=3 (20.0%) | FN n=1 (6.7%) | TN n=4 (26.7%) | FN n=0 (0.0%) | TN n=3 (20.0%) |
|  | **FDG** | | **FAPI** | | **Dual tracer** | |
| **Hepatocellular carcinoma** (n=7) | **Cancer** | **Benign** | **Cancer** | **Benign** | **Cancer** | **Benign** |
| **Positive PET/CT** | TP n=2 (28.6%) | FP n=2 (28.6%) | TP n=1 (14.3%) | FP n=0 (0.0%) | TP n=2 (28.6%) | FP n=2 (28.6%) |
| **Negative PET/CT** | FN n=0 (0.0%) | TN n=2 (28.6%) | FN n=1 (14.3%) | TN n=4 (57.1%) | FN n=0 (0.0%) | TN n=2 (28.6%) |

**Supplementary Table 2: Contingency tables for primary tumour analysis.** The total number of patients is based on the full study cohort minus those with an indeterminate scan result (n = 176 – 13 = 163). Dual-tracer PET/CT was deemed positive when at least one of the scans was positive for malignancy. *TP: True Positive, FP: False Positive, FN: False Negative, TN: True negative.*

| **Tumour Location** | **Radiotracer** | | | | | |
| --- | --- | --- | --- | --- | --- | --- |
|  | **FDG** | | **FAPI** | | **Dual tracer** | |
| **Involved lymph nodes** (n=76) | **Cancer** | **Benign** | **Cancer** | **Benign** | **Cancer** | **Benign** |
| Positive PET/CT | TP n=29 (38.2%) | FP n=11 (14.5%) | TP n=31 (40.8%) | FP n=1 (1.3%) | TP n=33 (43.4%) | FP n=11 (14.5%) |
| Negative PET/CT | FN n=11 (14.5%) | TN n=25 (32.9%) | FN n=9 (11.8%) | TN n=35 (46.1%) | FN n=7 (9.2%) | TN n=25 (32.9%) |
|  | **FDG** | | **FAPI** | | **Dual tracer** | |
| **Distant metastases** (n=30) | **Cancer** | **Benign** | **Cancer** | **Benign** | **Cancer** | **Benign** |
| Positive PET/CT | TP n=21 (70.0%) | FP n=1 (3.3%) | TP n=24 (80.0%) | FP n=2 (6.7%) | TP n=24 (80.0%) | FP n=2 (6.7%) |
| Negative PET/CT | FN n=5 (16.7%) | TN n=3 (10.0%) | FN n=2 (6.7%) | TN n=2 (6.7%) | FN n=2 (6.7%) | TN n=2 (6.7%) |

**Supplementary Table 3: Contingency tables for lymph nodal and distant metastases.** The total number of patients is based on the number of patients with available data on nodal (n=76) and distant metastases (n=30). Dual-tracer PET/CT was deemed positive when at least one of the scans was positive for malignancy. *TP: True Positive, FP: False Positive, FN: False Negative, TN: True negative.*

|  | **Tracer** | **Sensitivity (95% CI)**  **p-value*** | **Specificity (95% CI)**  **p-value*** | **PPV (95% CI)**  **p-value*** | **NPV (95% CI)**  **p-value*** | **Accuracy (95% CI)**  **p-value*** |
| --- | --- | --- | --- | --- | --- | --- |
| **Involved lymph nodes** (n=59) | FDG | 66.7% (47.8-81.4) | 68.8% (51.4-82.0) | 64.3% (45.8-79.3) | 71.0% (53.4-83.9) | 67.8% (55.1-78.3) |
|  | FAPI | 70.4% (51.5-84.1) | 96.9% (84.3-99.5) | 95.0% (76.4-99.1) | 79.5% (64.5-89.2) | 84.7% (73.5-91.8) |
|  | Dual tracer strategy | 77.8% (59.2-89.4) | 68.8% (51.4-82.0) | 67.7% (50.1-81.4) | 78.6% (60.5-89.8) | 72.9% (60.4-82.6) |
|  | FDG vs FAPI | 1.000 | **0.004** | 1.000 | **0.004** | **0.013** |
|  | Dual vs FDG | 0.250 | 1.000 | 0.250 | 1.000 | 0.250 |
|  | Dual vs FAPI | 0.500 | **0.004** | 0.500 | **0.004** | 0.065 |
| **Distant metastases** (n=12) | FDG | 55.6% (26.7-81.1) | 66.7% (20.8-93.9) | 83.3% (43.6-97.0) | 33.3% (9.7-70.0) | 58.3% (32.0-80.7) |
|  | FAPI | 66.7% (35.4-87.9) | 33.3% (6.1-79.2) | 75.0% (40.9-92.9) | 25.0% (4.6-69.9) | 58.3% (32.0-80.7) |
|  | Dual tracer strategy | 66.7% (35.4-87.9) | 33.3% (6.1-79.2) | 75.0% (40.9-92.9) | 25.0% (4.6-69.9) | 58.3% (32.0-80.7) |
|  | FDG vs FAPI | 1.000 | 1.000 | 1.000 | 1.000 | 1.000 |
|  | Dual vs FDG | 1.000 | 1.000 | 1.000 | 1.000 | 1.000 |
|  | Dual vs FAPI | 1.000 | 1.000 | 1.000 | 1.000 | 1.000 |

**Supplementary Table 4. Diagnostic accuracy of [^18^F]FDG, [^68^Ga]Ga-FAPI-46, and dual-tracer PET/CT for detecting lymph nodal and distant metastases in pathology-proven cases only.** Shown are the diagnostic accuracy metrics with their corresponding confidence intervals (CIs). Dual-tracer PET/CT was deemed positive when at least one of the scans was positive for malignancy. Red p-values represent a statistically significant decrease in diagnostic performance when using a dual-tracer approach compared to a single-tracer approach. Per patient analysis. *McNemar’s test. *PPV: Positive Predictive Value, NPV: Negative Predictive Value.*

| **Factor** | **OR FDG positive (95% CI)** | **p-value*** | **OR FAPI positive (95% CI)** | **p-value*** |
| --- | --- | --- | --- | --- |
| Chronic underlying liver disease (n = 6) | 1.01 (0.15-6.94) | 0.990 | 0.06 (0.06-0.54) | **0.013** |
| Chronic inflammatory pancreatitis (n = 12) | 1.26 (0.16-9.77) | 0.825 | 2.34 (0.19-28.53) | 0.503 |
| Biliary drain in situ (n = 48) | 1.12 (0.48-2.60) | 0.792 | 2.58 (0.68-9.83) | 0.165 |
| Previous surgical resection (n = 21) | 0.35 (0.12-1.01) | 0.052 | 2.18 (0.45-10.51) | 0.330 |
| Previous chemotherapy (n = 6) | 0.27 (0.04-1.67) | 0.159 | 0.30 (0.04-2.08) | 0.222 |

**Supplementary Table 5: Likelihood of a positive PET/CT result adjusted for case-mix.** * Logistic regression analysis. *OR: Odds Ratio, HCC: Hepatocellular Carcinoma.*

|  | **FDG PET/CT** | | | | **FAPI PET/CT** | | |
| --- | --- | --- | --- | --- | --- | --- | --- |
| **Factor** | **FP (%)** | **OR (95% CI)** | **p-value*** | **FP (%)** | | **OR (95% CI)** | **p-value*** |
| Chronic liver disease (n = 6) | 3 (50.0%) | 4.64 (0.79-27.20) | 0.089 | 2 (33.3%) | | 1.85 (0.28-12.41) | 0.528 |
| Chronic inflammatory pancreatitis (n = 12) | 3 (25.0%) | 1.98 (0.47-8.30) | 0.349 | 4 (33.3%) | | 2.87 (0.77-10.74) | 0.118 |
| Biliary drain in situ (n = 48) | 14 (29.2%) | 2.99 (1.28-7.01) | **0.012** | 14 (29.2%) | | 2.60 (1.12-5.99) | **0.025** |
| Previous surgical resection (n = 21) | 5 (23.8%) | 2.39 (0.72-7.95) | 0.156 | 7 (33.3%) | | 4.00 (1.34-11.93) | **0.013** |
| Previous chemotherapy (n = 6) | 0 (0.0%) | 0.0 (0.0-0.0) | 0.999 | 0 (0.0%) | | 0.0 (0.0-0.0) | 0.999 |

**Supplementary Table 6:** **Association between patient characteristics and false-positive PET/CT results.** * Logistic regression analysis. *FP: False Positive, OR: Odds Ratio.*

|  | **FDG PET/CT** | | | | **FAPI PET/CT** | | |
| --- | --- | --- | --- | --- | --- | --- | --- |
| **Factor** | **FN (%)** | **OR (95% CI)** | **p-value*** | **FN (%)** | | **OR (95% CI)** | **p-value*** |
| Chronic liver disease (n = 6) | 0 (0.0%) | 0.0 (0.0-0.0) | 0.999 | 1 (16.7%) | | 4.68 (0.44-49.98) | 0.202 |
| Chronic inflammatory pancreatitis (n = 12) | 1 (9.1%) | 0.52 (0.06-4.28) | 0.541 | 0 (0.0%) | | 0.0 (0.0-0.0) | 0.999 |
| Biliary drain in situ (n = 48) | 9 (18.8%) | 1.49 (0.61-3.65) | 0.380 | 1 (2.1%) | | 0.28 (0.03-2.40) | 0.247 |
| Previous surgical resection (n = 21) | 5 (25.0%) | 1.78 (0.55-5.74) | 0.336 | 0 (0.0%) | | 0.0 (0.0-0.0) | 0.999 |
| Previous chemotherapy (n = 6) | 2 (33.3%) | 2.11 (0.34-13.06) | 0.423 | 0 (0.0%) | | 0.0 (0.0-0.0) | 0.999 |

**Supplementary Table 7: Association between patient characteristics and false-negative PET/CT results.** * Logistic regression analysis. *FN: False Negative, OR: Odds Ratio.*

**Supplementary Figure 1: Receiver Operating Characteristic Curve for FDG SUV_max_.** Primary tumour FDG SUV_max_ was plotted against disease state (benign or malignant) according to the reference standard. The area under the curve was 0.591, indicating poor discriminatory performance.
